# Improved Strength Recovery and Reduced Fatigue with Suppressed Plasma Myostatin Following Supplementation of a *Vicia faba* Hydrolysate, in a Healthy Male Population

**DOI:** 10.3390/nu15040986

**Published:** 2023-02-16

**Authors:** Alish Kerr, Luke Hart, Heidi Davis, Audrey Wall, Seán Lacey, Andrew Franklyn-Miller, Nora Khaldi, Brian Keogh

**Affiliations:** 1 Nuritas Ltd., Joshua Dawson House, D02 RY95 Dublin, Ireland; 2 SSC Sports Medicine, Unit C10, Gulliver’s Retail Park, Northwood Avenue, Santry, D09 C523 Dublin, Ireland; 3 Research Integrity & Compliance Officer, Munster Technological University, T12 P928 Cork, Ireland

**Keywords:** bioactive peptide, muscle, strength, hydrolysate, performance, recovery, fatigue, *Vicia faba*, inflammation, exercise

## Abstract

Delayed onset muscle soreness (DOMS) due to intense physical exertion can negatively impact contractility and performance. Previously, NPN_1 (PeptiStrong™), a *Vicia faba* hydrolysate derived from a protein concentrate discovered through artificial intelligence (AI), was preclinically shown to help maintain muscle health, indicating the potential to mediate the effect of DOMS and alter molecular markers of muscle damage to improve recovery and performance. A randomised double-blind placebo-controlled trial was conducted on 30 healthy male (30–45 years old) volunteers (NCT05159375). Following initial strength testing on day 0, subjects were administered either placebo or NPN_1 (2.4 g/day). On day 14, DOMS was induced using resistance exercise. Strength recovery and fatigue were measured after 48 and 72 h. Biomarker analysis was performed on blood samples collected prior to DOMS induction and 0, 2, 48 and 72 h post-DOMS induction. NPN_1 supplementation significantly improved strength recovery compared to placebo over the 72 h period post-resistance exercise (*p* = 0.027), measured by peak torque per bodyweight, but not at individual timepoints. Muscle fatigue was significantly reduced over the same 72 h period (*p* = 0.041), as was myostatin expression (*p* = 0.006). A concomitant increase in other acute markers regulating muscle protein synthesis, regeneration and myoblast differentiation was also observed. NPN_1 significantly improves strength recovery and restoration, reduces fatigue and positively modulates alterations in markers related to muscle homeostasis.

## 1. Introduction

Muscle mass and sarcopenia are important factors when considering cardiometabolic health, cognitive function, the effect of anti-cancer therapies, as well as improved rehabilitation after injury or orthopaedic surgery and physical independence through ageing. While regular activity and resistance exercise are important parts of maintaining muscle health, exercise-induced muscle damage (EIMD) can occur following intense physical activity and resistance training [1]. This damage manifests as delayed onset of muscle soreness (DOMS) and can negatively impact people through muscle pain, reduced function and stiffness with a concomitant effect on recovery and performance [2]. Proposed mechanisms of DOMS include inflammation, calcium channel leakage, oxidative stressand muscle damage [3,4]. The cumulative effects of structural and systemic characteristics of DOMS can be recorded for up to 7 days post-exercise, with peak effects seen between 24 and 96 h [5]. Hence, there is significant scope to shorten this window.

Reducing the effects of DOMS is confounded by the molecular complexity, making interventions challenging. Nutritional intervention strategies have had some success addressing biomarker expression of DOMS [6]. Examples of successful interventions include protein supplementation, such as whey, which suppressed post-exercise increases in IL-6 levels in a female sarcopenic population, following 12 weeks of treatment [7], and omega-3 polyunsaturated fatty acids (eicosapentaenoic acid and docosahexaenoic acid), which significantly decreased circulating IL-6 and creatine kinase (CK) levels at 24 h post-isokinetic testing compared to pre-supplementation in endurance athletes [8]. Recently, using a countermovement jump protocol, a lemon verbena extract was shown to reduce muscle damage and improve recovery compared to placebo, post-EIMD, although inflammatory markers did not differ with treatment [9]. Similar benefits have been seen with tart Montmorency cherry promoting recovery and attenuating IL-6 and C-reactive protein expression following EIMD in trained cyclists [10]. In a healthy male population, 20 g/day of creatine, over 6 days, was shown to reduce muscle soreness and spikes in CK, while also improving range of motion following repeat resistance exercise sessions [11]. Additionally, an 8-day supplementation of pomegranate juice (650 mg gallic acid equivalents (GAE)/day and 1300 mg GAE/day) significantly improved recovery over 96 h, following eccentric exercise performed on day 4 of supplementation, in a non-resistance-trained healthy male population [12]. As these studies have shown that nutritional supplementations have exerted beneficial effects post-exercise, accordingly, we present a peptide application, where characterised bioactive peptides with defined activity are contained within a peptide network to affect relevant areas for muscle health function [13,14].

Characterising bioactive peptides in a nutrient-dense food source is a time-consuming and serendipitous endeavour, with multiple fractionation steps required [15]. Artificial intelligence (AI) offers the possibility of deciphering the dense molecular network within food, with the additional benefit of targeted discovery for a specific health need [15]. Recently, AI and machine learning (ML) techniques have identified active peptide networks/hydrolysates with characterised constituent key bioactive peptides in areas such as inflammation [16] and glucose regulation [17]. In line with this approach, AI and ML techniques were used to identify bioactive peptides which could address muscle protein synthesis, muscle breakdown and inflammation [18]. Among predicted peptides, two peptides were shown to significantly increase protein synthesis (histidine–leucine–proline–serine–tyrosine–serine–proline–serine–proline–glutamine; HLPSYSPSPQ) and reduce pro-inflammatory cytokine release (threonine–isoleucine–lsyine–isoleucine–proline–alanine–glycine–threonine; TIKIPAGT) in vitro. These efficacious peptides were identified in a hydrolysate derived from *Vicia faba* protein concentrate, NPN_1 (PeptiStrong™), as previously described by Corrochano et al., 2020 [18]. NPN_1 has been shown to address multiple aspects related to muscle health, including increased muscle protein synthesis, reduced tumour necrosis factor-alpha (TNF-α) secretion in vitro and reduced expression of genes associated with muscle atrophy [19]. In a hindlimb suspension murine model, following 18 days of NPN_1 supplementation, treated mice exhibited significantly reduced muscle loss in the suspended soleus muscle, increased mitochondrial biogenesis and myogenesis markers, as well as enhanced integrated density of type I and II muscle fibres [19]. Additionally, both constituent bioactive peptides (HLPSYSPSPQ and TIKIPAGT) contained within NPN_1 were shown to survive simulated gastrointestinal digestion with the potential to transfer across the lumen into the blood vessels. These peptides demonstrated adequate stability following in vitro incubation with human plasma, which may correlate to health benefits observed preclinically [18].

Previously, we have shown that NPN_1 induced an increase in phosphorylated S6 with a concomitant decrease in atrophy associated genes in vitro, with the effects translating into a benefit in a murine model [19]. In the present study, the objective was to investigate the effect of NPN_1 on strength recovery in a double-blind, placebo-controlled clinical trial in healthy male volunteers. Secondary to this, we also measured expression of a range of plasma myokines in both groups. We hypothesised that NPN_1 supplementation would have a beneficial effect on EIMD, and hence promote strength recovery.

## 2. Materials and Methods

### 2.1. Subjects

Subjects were recruited from internal databases at the study site, advertisements on social media, and notice boards in public buildings for a double-blind, placebo-controlled clinical trial in healthy male volunteers. A male population was chosen due to cohort availability. One hundred forty-two male subjects responded to the advertising campaign and received detailed information about the study. From these, seventy-six subjects were eligible for pre-screen. This trial was not powered, as this was a pilot trial, and population size was chosen based on similar studies carried out with dietary supplements. Thirty healthy, non-smoking, moderately active (exercise 1–3 times per week) males aged between 30 and 45 years with a BMI between 18 and 30 kg/m^2^ met the inclusion criteria. Detailed inclusion and exclusion criteria are presented in Table 1. Eligibility was evaluated by physical examination and interview with a consultant physician. All subjects conducted a COVID-19 exposure questionnaire and signed an informed consent form prior to any procedures, having been provided the information a week prior to consent.

### 2.2. Trial Design

**Figure 1:**
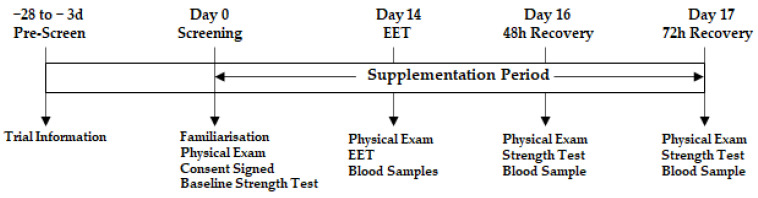
General schematic of trial design.

This study was a double-blind, randomised parallel group trial that investigated the effects of NPN_1 supplementation on muscle strength and recovery after exhaustive exercise (NCT05159375; (www.clinicaltrials.gov; accessed on 16 December 2021), registered retrospectively). After inclusion, subjects were randomly allocated to either placebo (silicified microcrystalline cellulose; SMCC) or NPN_1 supplementation; randomisation was carried out using blocking (blocks of 4) according to the statistical analysis plan, by an unblinded contact in Nuritas Ltd. (AW, Dublin, Ireland). All participants and researchers were blinded for the duration of the study. Subjects were instructed to ingest the supplement with their first meal of the day. Baseline strength measurements were taken prior to supplementation. Fourteen days post-supplementation, EIMD was performed to induce DOMS. Strength measurements were repeated at 48 h and 72 h post-EIMD exhaustive exercise routine. Venous blood samples were obtained prior to commencement of the DOMS-inducing exercise routine and 0, 2, 48 and 72 h following completion of the routine. A graphical illustration of the trial design is displayed in Figure 1. This trial was conducted in compliance with the Declaration of Helsinki and ethical approval was granted by the Institutional Review Board “Sports Surgery Clinic Research Ethics Committee” (PN20.004.01). The study was performed from August 2021 to February 2022 at the Sports Surgery Clinic, Santry, Dublin, an independent study site that is focused on sports medicine.

### 2.3. Ingredient Production and Supplementation

NPN_1 (PeptiStrong™) is a proprietary ingredient derived from *Vicia faba* powdered protein concentrate, available upon request from Nuritas Ltd., and was produced according to Cal et al. (2020) for the specific purpose of this trial. Here, *Vicia faba* protein concentrate was homogenised in solution. Hydrolysis was achieved with a food-grade endoprotease controlling for enzyme-specific conditions, such as temperature and pH value (approximately pH 6). Enzymatic inactivation was achieved by raising the temperature to 85 °C; the solution was spray-dried utilising a standard spray-drying process at air inlet temperatures above 160 °C [19]. All batches of NPN_1 underwent peptidomics analysis using LC-MS/MS, outlined in Corrochano et al., 2021 [18]. Here, batches were correlated based on peptidomic profiles and to verify the presence of characterised constituent bioactive peptides which have previously been synthesised and validated in vitro for bioactivity, such as histidine–leucine–proline–serine–tyrosine–serine–proline–serine–proline–glutamine (HLPSYSPSPQ) and threonine–isoleucine–lsyine–isoleucine–proline–alanine–glycine–threonine (TIKIPAGT) [18]. SMCC was used as a placebo. NPN_1 and placebo were formulated into hydroxypropyl methylcellulose (HPMC) capsules of the same colour and size. Subjects were instructed to take 5 capsules daily, equating to a 2.4 g serving of NPN_1.

### 2.4. Strength Measurements

Strength measurements, as a primary endpoint, were taken at baseline (day 0), day 16 and day 17. Height and body mass were measured immediately prior to testing (Portable SECA 213 Stadiometer). All participants completed a warm-up consisting of 5 min cycle ergometer (Wattbike). Subjects then underwent concentric knee extension and flexion strength testing, assessed at an angular velocity of 60°/s through the range of 0–100° knee flexion using an isokinetic dynamometer (Cybex NORM; Computer Sports Medicine Inc, Stoughton, MA, USA). High relative reliability and moderate absolute reliability have been found for this protocol using the Cybex NORM [20,21], and an angular velocity of 60°/s has been found to identify the greatest strength deficits [22,23]. The participants performed a warm-up set of five repetitions of knee extension and flexion, building up from 60% to 100% of maximal effort. After a 60 s rest period, the participants completed two maximal effort sets of 5 repetitions, with a 60 s rest period between each set. They were instructed to push and pull as hard and fast as possible against the resistance with verbal encouragement. The non-dominant limb was tested first before repeating the procedure with the dominant limb.

### 2.5. Exhaustive Exercise Test (EET)

EET was performed on day 14 of supplementation. Height and body mass were measured immediately prior to testing. All participants completed a warm-up consistent with the strength measurements regime. Following the warm-up and a 90 s rest period, the participants completed five maximal effort sets of 8 repetitions, with a 90 s rest period between each set. Subjects were given verbal instructions to push and resist while performing the EET. The non-dominant limb was tested first before repeating the procedure with the dominant limb.

### 2.6. Fatigue Index (FI)

FI was calculated as FI = [(highest force − lowest force)/(highest force)] [24]; this exploratory analysis was calculated from strength measurements already recorded by participants. Highest force was calculated as the average torque from all repetitions from the initial maximal effort set on the isokinetic dynamometer. Lowest force was calculated as the average torque from all repetitions from the final maximal effort set on the isokinetic dynamometer.

### 2.7. Myokine Array

The MILLIPLEX multiplex assays (Merck, Darmstadt, Germany) using xMAP technology (Luminex Corporation, Austin, TX, USA) was used to analyse the concentrations of various analytes within the plasma samples. MILLIPLEX magnetic beads panels (Merck) were used to analyse 14 myokines. A full list of analytes can be found in Supplementary Table S1. All procedures were performed according to the manufacturer guidelines. Standard curves were created for each analyte, using standard concentrations depending on manufacturer guidelines for each panel. Two quality controls provided in the kits were added to each panel. Analysis of Luminex panels was performed using the Luminex 200 (Luminex Corporation) instrument; for acquisition, the xPONENT software (v.3.1.7; Luminex Corporation) was used. The median fluorescent intensity was analysed using a 5-parameter logistic curve-fitting to calculate the concentration of analytes in each sample.

### 2.8. Data Analysis and Statistics

The analysis objective was to observe differences in the strength recovery, fatigue index and plasma markers between placebo and NPN_1 supplementation. Adjudication of trial adherence was performed blinded; those who did not adhere to the inclusion/exclusion criteria throughout the trial were removed from the per protocol analysis group.

Data were analysed using GraphPad Prism Version 9. Descriptive statistics are presented numerically in terms of the mean ± SEM, and graphically using error bar plots and boxplots. All statistical tests were performed two-sided and interpreted using a 5% level of significance. For exploratory purposes, appropriate additional tests were used to determine between- and within-group differences. Where appropriate, a ROUT outlier analysis was performed with a 1% threshold [25]. Where appropriate, data satisfied the conditions of normal distribution and homogeneity of variances (confirmed with the appropriate plots and using Shapiro–Wilk and Levene tests, respectively). For the evaluation of treatment effects on strength recovery over the course of the study, a repeated measures ANOVA and incremental area under the curve (iAUC) analysis were performed. For the evaluation of the treatment on strength recovery at each time point, Student’s *t*-test was performed. Strength recovery was calculated relative to each subject’s baseline strength test pre-supplementation, expressed as peak torque/body weight. For the evaluation of treatment effects on FI and serum plasma markers over the course of the study, a repeated measures ANOVA was performed. For the evaluation of the treatment on FI and serum plasma markers at each time point, if normal distribution was satisfied, Student’s *t*-test was performed; otherwise, a Mann–Whitney test was performed. Due to explorative data analysis, no correction for multiple comparison was performed. The results presented below refer to the per protocol data set (PP). PP criteria were pre-defined in the protocol: missing data, adverse events or use of prohibited concomitant medication interfering with study results, and major protocol violations. As stated above, adjudication to determine adherence to the protocol was performed blindly and agreed upon by four adjudicators.

## 3. Results

### 3.1. Trial Design

**Figure 2:**
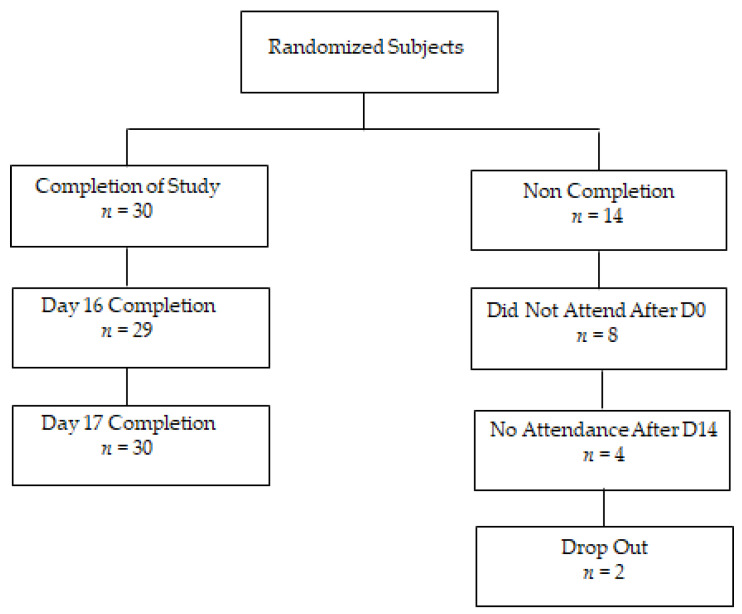
Recruitment flow chart.

A total of 44 subjects were randomised for this trial, of which 30 completed the study (Figure 2). Eight subjects did not attend following the initial screen visit at day 0. Four subjects withdrew following the exercise session on Day 14 and two subjects did not complete the protocol.

Deviations from protocol are outlined in Table 2. If a subject exercised within 48 h of the initial exercise session on Day 14 and recorded a score of 13 or higher on the Borg Scale, they were excluded from the PP population.

Five participants were excluded from the PP population. Anthropometric data are outlined in Table 3. Age, height, body mass and BMI were evenly distributed between the two treatment groups. There were no serious adverse events reported. Non-serious adverse events included two reports of muscle tightness on isokinetic dynamometry, one case of high blood pressure which settled post-testing with no issues, and one case of total body DOMS which prevented participation in Day 16 testing. This subject recovered fully with no pain 48 h following Day 17.

### 3.2. Strength Recovery

**Figure 3:**
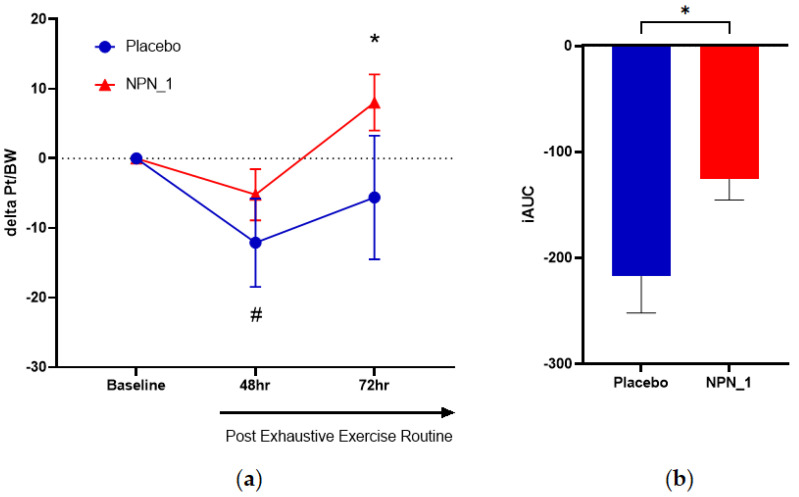
NPN_1 group had significantly increased strength recovery post-strenuous exercise over the hour period post-resistance exercise, but not at individual timepoints. (**a**) Delta peak torque per bodyweight was calculated per subject from their own baseline and expressed over time (mean ± SEM, RM-ANOVA, *p* = 0.226). * Indicates NPN-1 is significantly different from baseline strength (*t*-test, *p* = 0.025). # Indicates Placebo is significantly different from baseline strength (*t*-test, *p* = 0.032). (**b**) iAUC of strength recovery was calculated over the study duration (mean ± SEM, *t*-test, *p* = 0.020).

Peak torque per bodyweight, the measure of muscle strength, was obtained at baseline (pre-supplementation), 48 h (16 days of supplementation) and 72 h (17 days of supplementation) after the exhaustive exercise routine. Inter-group analysis for pre- and post-EIMD showed that muscle strength was significantly reduced (*p* = 0.032) from baseline in the placebo group at 48 h, whereas no significant reduction in strength was observed in the NPN_1-supplemented group. By 72 h, the NPN_1 cohort showed a significant increase (*p* = 0.025) in strength from baseline values, whereas the placebo cohort did not (Figure 3a). Additionally, subjects who received NPN_1 recovered to baseline values within 48 h and increased significantly higher (*p* = 0.025) than baseline values at 72 h. However, subjects supplemented with placebo displayed a significantly (*p* = 0.032) lower recovery from baseline values at 48 h and still had not fully recovered to baseline within 72 h. To assess the effect between groups over the test period, an iAUC analysis was carried out for each participant leg, followed by ROUT analysis, with a 1% threshold (Figure S1). While the total number of participants in each analysis did not change (Placebo, N = 10; NPN_1, N = 14), single data points for three participants within the NPN_1 group were removed. As shown in Figure 3b, NPN_1-supplemented subjects showed a significant increase (*p* = 0.020) in strength recovery compared to placebo over the 72 h period post-resistance exercise, while no significant change was observed at individual timepoints.

### 3.3. Fatigue Index

**Figure 4:**
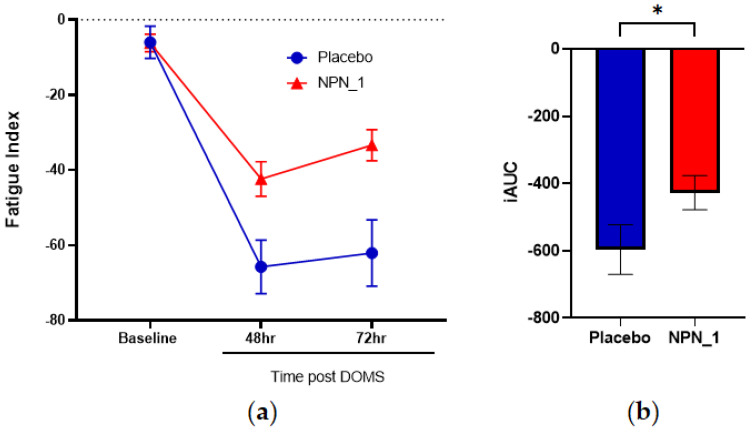
NPN_1 significantly relieves fatigue and allows a return to performance. (**a**) Fatigue index was calculated between sets at each strength measurement timepoint (mean ± SEM, RM-ANOVA, *p* = 0.002) and expressed over time. Significant effects were also observed at individual time points of 48 h. (**b**) iAUC of fatigue index was calculated over the study duration (mean ± SEM, Mann–Whitney, *p* = 0.041, * *p* < 0.05).

FI was determined by measuring the difference in mean torque of the repetitions performed in the maximal effort sets at baseline, 48 and 72 h post-exhaustive exercise routine. No differences were observed in baseline FI values between groups. Subjects supplemented with NPN_1 showed a significant (*p* = 0.002) benefit on FI (Figure 4a), leading to a significant performance benefit over the 72 h period post-DOMS induction, compared to placebo (*p* = 0.041; Figure 4b).

### 3.4. Molecular Markers of Muscle Recovery

**Figure 5:**
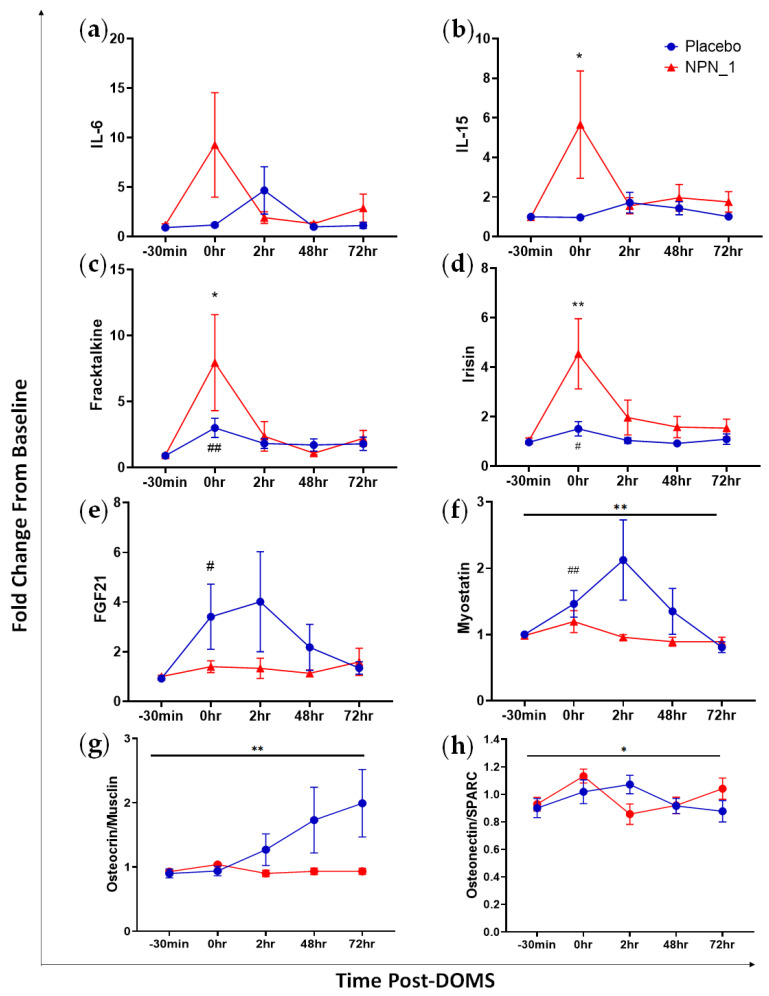
NPN_1 supplementation alters plasma myokine concentrations, increasing myokines associated with glycogenesis and protein synthesis, whilst decreasing those associated with negative regulation of protein synthesis. Serum myokine expression following intense exercise is altered with supplementation with NPN_1. Analysed by repeated measures ANOVA. (**a**) Effect of supplementation on IL-6 (mean ± SEM, RM-ANOVA, treatment effect: *p* = 0.138). (**b**) IL-15 (mean ± SEM, RM-ANOVA, treatment effect: *p* = 0.159, NPN_1 change from baseline, *p* = 0.045). (**c**) Fractalkine (mean ± SEM, RM-ANOVA, treatment effect: *p* = 0.351, NPN_1 change from baseline, *p* = 0.030, Placebo change from baseline, *p* = 0.003). (**d**) Irisin (mean ± SEM, RM-ANOVA, treatment effect: *p* = 0.096, NPN_1 change from baseline, *p* = 0.009, Placebo change from baseline, *p* = 0.031). (**e**) FGF21 (mean ± SEM, RM-ANOVA, treatment effect: *p* = 0.0662, Placebo change from baseline, *p* = 0.027). (**f**) Myostatin (mean ± SEM, RM-ANOVA, treatment effect: *p* = 0.006, Placebo change from baseline, *p* = 0.010). (**g**) Osteocrin/Musclin (mean ± SEM, RM-ANOVA, treatment effect: *p* = 0.009). (**h**) Osteonectin/SPARC (mean ± SEM, RM-ANOVA, treatment effect: *p* = 0.025). Black line indicates significant treatment effect. * (*p* < 0.05)/** (*p* < 0.01) Indicates significant change from baseline values with NPN_1 supplementation following DOMS induction. # (*p* < 0.05)/## (*p* < 0.01) Indicates significant change from baseline values with placebo supplementation following DOMS induction.

A myokine array was performed for a range of biomarkers associated with muscle health on serum samples from subjects 30 min before exhaustive exercise on Day 14 (−30 min), immediately following exhaustive exercise (0 h); 2 h following exhaustive exercise (2 h); 48 h following exhaustive exercise; Day 16 (48 h); and Day 17 (72 h). Eight analytes of interest are shown in Figure 5; the results for the remaining myokines are shown in Figure S2. Expression of IL-6 was higher at the 0 h timepoint following NPN_1 supplementation compared to placebo (Figure 5a). IL-6 concentrations did not differ significantly over the course of the study (*p* = 0.138) and returned to baseline values within 48 h. Similarly, the concentration of IL-15 was elevated following NPN_1 supplementation compared to placebo, although not significantly over time (*p* = 0.159; Figure 5b); values for both cohorts returned to baseline within 2 h post-exhaustive exercise. Interestingly, IL-15 release was significantly higher immediately post-DOMS compared to baseline (−30 min) within the NPN_1 treatment group, with no treatment effect seen. Fractalkine and irisin exhibited similar pre/post-DOMS induction profiles within each group (Figure 5c,d). Here, release was transiently increased significantly immediately post-DOMS for both groups for fractalkine (NPN_1, *p* = 0.030; Placebo, *p* = 0.003) and irisin (NPN_1, *p* = 0.009; Placebo, *p* = 0.031). All groups returned to baseline values within 2 h following exhaustive exercise. FGF21 release was reduced following DOMS induction in the NPN_1 supplementation group; however, this was not statistically different over the course of the study (*p* = 0.066; Figure 5e). Of note, subjects within the placebo-supplemented arm recorded significantly higher than baseline values immediately post-DOMS (*p*= 0.0266). Myostatin release was significantly inhibited in the NPN_1-supplemented arm compared to placebo over the course of the study (*p* = 0.006), whereas the placebo group exhibited significantly higher myostatin release at 0 h compared to baseline values (Figure 5f). The placebo treatment arm displayed a significant increase in osteocrin/musclin release over the course of the study compared to NPN_1 treatment (*p* = 0.009; Figure 5g). A significantly higher treatment effect on osteonectin/SPARC was seen with NPN_1 treatment compared to placebo (*p* = 0.025; Figure 5h).

## 4. Discussion

In this study, NPN_1 supplementation improved strength recovery, reduced fatigue and suppressed myostatin expression in a healthy male population following EIMD. Previously, we have shown that NPN_1 induced an increase in phosphorylated S6 with a concomitant decrease in atrophy-associated genes in vitro [19]. Thus, we hypothesised that NPN_1 supplementation would have a beneficial effect on exercise-induced muscle damage, and hence promote strength recovery. Following induction of DOMS, a decrease in strength was observed in both cohorts. A significant (*p* = 0.020) recovery in muscle strength was observed following NPN_1 supplementation compared to placebo over the 72-h period post-resistance exercise. Fatigue was significantly decreased (*p* = 0.041) over the same 72-h period in the NPN_1 group compared to placebo. Additionally, the release of myostatin post-DOMs was beneficially modulated (*p* = 0.006) in the NPN_1-supplemented group compared to placebo. As a reduction in muscle damage and fatigue is known to protect muscle post-strenuous exercise [26,27], these cumulative results indicate the potential for NPN_1 to reduce the severity of DOMS and improve recovery, hence allowing a faster return to training.

Exploratory iAUC analysis showed a 54% improvement in performance of isokinetic leg extension with NPN_1 supplementation compared to placebo over the 72 h after EIMD. NPN_1 supplementation significantly increased muscle strength recovery (*p* = 0.027), and accordingly, a full recovery of strength was recorded within the window when the peak effects of DOMS are typically observed (24–96 h) [5]. Different methods to induce muscle damage are used for different nutritional intervention studies. A similar EIMD protocol carried out with tart cherry (120 g/day TartVitaCherry^®^) in a healthy male and female population showed no significant differences for strength recovery [28], while other types of muscle damage induction have shown beneficial effects on strength recovery with tart cherry [10,29] and other nutritional supplements, such as lemon verbena [9]. In a previous study in resistance-trained individuals with several years of experience, supplementation with branched-chain amino acids (BCAA) for eight days at 0.22 g/kg/day showed no effect on muscle function after eccentric EMID; however, they did record a decrease in muscle soreness, indicating the importance of perceived benefits to supplementation [30]. In the current study, subjects within the NPN_1-supplemented arm recovered fully within 48 h and exceeded baseline values at 72 h. In contrast, the placebo-supplemented arm had lower recovery values compared to baseline at 48 h and had still not fully returned to baseline following 72 h. Resistance-type exercise is known to increase muscle protein synthesis through activation of mTOR for up to 36 h, with repeated exercise activating ribosomal activation and total RNA (ribonucleic acid) content [31]. Importantly, increased muscle protein synthesis has been shown to help induce muscle repair [32]. Previously, NPN_1 was shown to induce expression of genes involved in myogenesis (mTOR and MYF5) in a murine model of atrophy as well as p-S6 expression in vitro [18,19]. These observations indicate that NPN_1 would be a candidate for use in muscle repair or adaptation in vivo. We recently concluded a study to investigate the effects of NPN_1 supplementation on short-term immobilisation and subsequent recovery [33]. In the study, healthy males received either 10 g of NPN_1 or milk protein concentrate (MPC) twice daily whilst subjected to 7 days of one-legged knee immobilisation followed by 14 days of ambulant recovery. NPN_1 performed similarly to MPC for recovery of muscle mass and strength; however, subjects supplemented with NPN-1 regained muscle strength to the level measured at baseline, whereas the subjects supplemented with MPC did not. An important indicator of the balance between protein synthesis and protein breakdown is the muscle fractionate synthetic rate (FSR). An increase in FSR indicates that protein synthesis supersedes the rate of protein breakdown and is a measurement of muscle conditioning [34]. Of key clinical interest was the finding that NPN_1 significantly outperformed MPC in FSR, indicating a possible benefit for anabolic pathways and a possible shorter recovery period. While plant proteins have been shown to increase FSR but only to the same level as milk [35], this is a highly significant finding for a plant protein source to outperform an animal one. The increase in FSR observed with NPN_1 is not observed with the raw unhydrolysed material, indicating that the effect is mediated by the AI-predicted bioactive peptides [36]. Consequently, in line with previous studies, a peptide-specific benefit within NPN_1 supplementation for muscle protein synthesis may help stimulate strength recovery and improve recovery beyond baseline post-EIMD. This is further supported in the current study, as administration of 2.4 g of NPN_1 would not elicit nutrition-associated anabolic effects without peptide-specific signalling events on relevant pathways, and subjects would have sufficient dietary protein.

Muscle fatigue is a major symptom of DOMS. The fatigue index measurements at 48 and 72 h post-EIMD were calculated using the highest and lowest force recorded during these sets [24]. Following induction of DOMS, fatigue was significantly attenuated over each set in the NPN_1-supplemented arm compared to placebo. The reduced fatigue index recorded with NPN_1 supplementation is in line with the improved strength recovery values compared to baseline. Similarly, lemon verbena supplementation exhibited reduced fatigue; this was not calculated through a fatigue index, they inferred the benefit through attenuated maximal voluntary contraction following resistance-type exercise and complete recovery within 48 h following EIMD [9]. This study also induced DOMS differently, using a countermeasure jump protocol, and may introduce variability in DOMS induction. Importantly, the severity and consistency of exercise-induced DOMS is dependent upon the method used to induce it. Many studies use free exercise, e.g., squats or counter-jumps, to induce DOMS. These add variability from subject to subject as there is less control versus isokinetic dynamometry, which brings greater consistency to our study for DOMS induction. A major contributory factor to muscle fatigue experienced during DOMS is impaired calcium release [37]. Interestingly, NPN_1 supplementation was previously shown to increase expression of PPP3CA in a murine disuse model [19], which is induced through elevated calcium release and results in the expression of calcineurin and is an important factor in muscle regeneration following injury [38]. Additional biomarkers such as those for ATP metabolism and ROS could be beneficial to investigate to further elucidate the mechanisms involved in the improved fatigue index effect following NPN_1 supplementation.

Myokines are cytokines released by muscle cells in response to muscular contractions [39], the measurement of which in the plasma can give an indication of injury to muscle tissue. We used a myokine array to investigate the effect of NPN_1 supplementation on muscle strength recovery. We observed that several myokines were beneficially modulated immediately after the induction of EIMD in the NPN_1 group. Of note, we recorded an increase in irisin expression, which can induce glycogenesis [40], as well as a significant increase in IL-15 (*p* = 0.159), which has been linked to increased muscle mass and can promote myoblast differentiation [41]. This glycogen replenishment and possible muscle regeneration may, in part, be responsible for the improved strength recovery and the fatigue index recorded following NPN_1 supplementation. The myostatin–Smad2/3 pathway is a major signalling pathway for protein synthesis, where myostatin acts as a negative regulator [42]. Myostatin was significantly suppressed in the NPN_1 group compared to placebo over the course of the trial, as was the release of fibroblast growth factor 21 (FGF21) in the NPN_1 group at 0 and 2 h. These combined data indicate clinical evidence of attenuation of muscle breakdown with NPN_1 supplementation [43,44], albeit further work is required to identify the minimal clinically important difference in muscle strength recovery and NPN_1 supplementation in a powered trial. In this respect, it is difficult to compare the effect of NPN_1 supplementation on myostatin to other nutritional interventions such as tart cherry, pomegranate and lemon verbena, as many studies into effects on EIMD focus on inflammatory and oxidative stress biomarkers rather than muscle health biomarkers [9,45,46]. It is known that acute transient inflammation promotes healing of healthy skeletal muscle [47]. For example, IL-6 is a major regulator of myogenesis, and acute IL-6 expression can increase protein synthesis, satellite cell proliferation and can lead to an anti-inflammatory signalling cascade [47]. In similar previous studies, tart cherry [10,29] and omega-3 polyunsaturated fatty acids [8] were shown to have an overall treatment effect on attenuating IL-6 compared to placebo, whereas lemon verbena [9] and pomegranate did not produce treatment effects [45]. Interestingly, in the present study, IL-6 expression was transiently increased at 0 h post-EIMD in the NPN_1 group, which may aid in the improved strength recovery observed. As changes return to baseline quite quickly, the likely source of IL-6 is the myocyte, as opposed to an immune cell release of IL-6. The upregulation of myokines such as fractalkine, osteocrin/musclin and osteonectin/SPARC has been shown to play a role in regeneration, mitochondrial biogenesis and adaption of muscle to exercise [39,48,49]. The significant increase in expression of fractalkine (*p* = 0.030) and osteonectin/SPARC (*p* = 0.025) in the NPN_1 group may contribute to the reduced fatigue experienced following EIMD. Additionally, another important finding was the significantly higher expression of osteocrin/musclin (*p* = 0.009) in the placebo treatment arm compared to NPN_1. This contrast may indicate a mechanism of action for NPN_1 independent of osteocrin/musclin, which is an important factor to consider in future studies.

As predicted using AI, the in vitro effects observed for NPN_1 [19] have translated into a clinical benefit for strength recovery. Of note, this characterised ingredient with cell-specific signalling for protein synthesis and anti-inflammatory effects could be used in a complimentary supplement approach with other efficacious ingredients. NPN_1 supplementation may thus serve to achieve a balance between muscle protein synthesis, muscle breakdown and inflammation, inducing a quicker return to muscle homeostasis post-EIMD.

A limitation of the current study would be the comparison of NPN_1 to SMCC rather than an unhydrolysed *Vicia faba* protein, which should be addressed in future studies. An additional limitation to note is the different physiological response of male and female subjects to nutritional interventions. For example, a gender effect on blood lactate was seen in subjects following supplementation with mango leaf extract during repeated sprint exercises [50]. Therefore, it would be of interest in future studies to assess the effects of NPN_1 in a female cohort.

## 5. Conclusions

NPN_1 is a characterised plant-based efficacious ingredient that we have shown at low dose to improve strength recovery and reduce fatigue following strenuous activity. In this trial, we have further shown that NPN_1 supplementation altered the plasma concentration of myokines associated with muscle health and/or glycogen metabolism, with a subsequent benefit for strength recovery. As such, NPN_1 represents a potential supplement to promote faster recovery following strenuous activity.

## Figures and Tables

**Table 1:** Subject inclusion and exclusion criteria.

Inclusion Criteria	Exclusion Criteria
Males between 30 and 45 years of age. Participants agree to abstain from taking additional supplements throughout the testing period, with particular emphasis placed upon protein-based products. Participants agree to maintain their normal diet and exercise routine throughout the study. BMI between 18.5 and 29.9 kg/m^2^. Participants agree to refrain from consuming alcohol in the 48 h leading up to a test day. Willingness to complete questionnaires, records and diaries associated with the study and to complete all clinic visits. Provide voluntary, written, informed consent to participate in the study. Refrain from any sort of exhaustive physical exercise from 48 h prior to each test or blood draw. Healthy as determined by medical examination at screening visit. Willingness to complete food diaries during the study. Must have a smart phone to use the Nutritics App. Non-smoker.	Alcohol or drug abuse in past year. Participation in any other clinical trial in the past 3 months from time of randomisation. Volunteer has a known allergy to the test material’s active or inactive ingredients. Volunteers with unstable medical conditions. Any complaints that could interfere with ability to exercise. Individuals who are cognitively impaired and/or who are unable to give informed consent. Any co-morbidities interacting with mobility or muscle metabolism of the lower limbs (e.g., arthritis, spasticity/rigidity, all neurological disorders, paralysis). Creatine supplements, anticoagulants, corticosteroids, growth hormones. Presence or history of neurological disorders or significant psychiatric illness. Any other condition which in the investigator’s opinion may adversely affect the volunteer’s ability to complete the study or its measures or which may pose significant risk to the volunteer. Participation in resistance or aerobic exercise within 48 h of the test days. Participation in >3 high-intensity exercise sessions per week. Undertake no recovery methods such as sea swims, foam rolling, cryotherapy or undue stretching during Days 14–17. Have been in contact with a suspected or confirmed case of COVID-19 in the previous 14 days. Are Hepatitis A- or B-positive, HIV-positive or have had a sexual partner who is infected with hepatitis or HIV.

**Table 2:** Population allocation adjudication results based on exclusion criteria.

Participant ID	Group	Issue	Adjudicated Result
7	Placebo	Exercise performed within 48 h of test day. Borg Scale ≤ 13	Include in PP
9	Placebo	Exercise performed within 48 h of test day. Borg Scale > 13	Exclude from PP
12	Placebo	Exercise performed within 48 h of test day. Borg Scale > 13	Exclude from PP
15	Placebo	Exercise performed within 48 h of test day. Borg Scale > 13	Exclude from PP
38	Placebo	Exercise performed within 48 h of test day. Borg Scale ≤ 13	Include in PP
40	Placebo	Exercise performed within 48 h of test day. Borg Scale > 13	Exclude from PP
8	NPN_1	Exercise performed within 48 h of test day. Borg Scale ≤ 13	Include in PP
10	NPN_1	Exercise performed within 48 h of test day. Borg Scale ≤ 13	Include in PP
11	NPN_1	Exercise performed within 48 h of test day. Borg Scale ≤ 13	Include in PP
22	NPN_1	Exercise performed within 48 h of test day. Borg Scale > 13	Exclude from PP

**Table 3:** Anthropometric data (mean ± SD).

	Placebo (*n* = 10)	NPN_1 (*n* = 14)
Age (years)	38 ± 4.8	37.1 ± 5.0
Height (cm)	180.8 ± 5.8	182.9 ± 7.1
Body Mass (kg)	86.3 ± 11.0	85.8 ± 9.9
BMI (kg/m^2^)	26.3 ± 2.3	25.6 ± 2.4

## Data Availability

The data presented in this study are available on request from the corresponding author. The data are not publicly available due to confidentiality.

## Supplementary Materials

The following supporting information can be downloaded at: https://www.mdpi.com/article/10.3390/nu15040986/s1, Table S1: Myokine analytes; Figure S1: Outlier analysis; Figure S2: Serum myokine expression following intense exercise with supplementation with NPN_1.
