# Supplementary material for: Improved Strength Recovery and Reduced Fatigue with Suppressed Plasma Myostatin Following Supplementation of a Vicia faba Hydrolysate, in a Healthy Male Population
Source: Nutrients. 2023 Feb 16;15(4):986. doi: 10.3390/nu15040986 (PMC9967853; doi:10.3390/nu15040986)
Supplement: Supplementary file 1 [file nutrients-15-00986-s001.zip › nutrients-2186777-supplementary.pdf]

Table S1. List of myokine analytes investigated.

**Myokine Analytes**	
Brain-derived neurotrophic factor (BDNF)	Irisn
Erythropoietin<br>(EPO)	Leukemia inhibitory factor<br>(LIF)*
Fatty Acid Binding Protein 3 (FABP3)	Myostatin/GDF8
Fibroblast Growth Factor 21<br>(FGF-21)	Oncostatin-M<br>(OSM)*
Fractalkine/CX3CL1	Osteocrin (OSTN)/Musclin
Follistatin-like 1<br>(FSTL1)	Osteonectin/SPARC
Interleukin 6<br>(IL-6)	Interleukin 15<br>(IL-15)

**Myokines not represented due to inconclusive results*

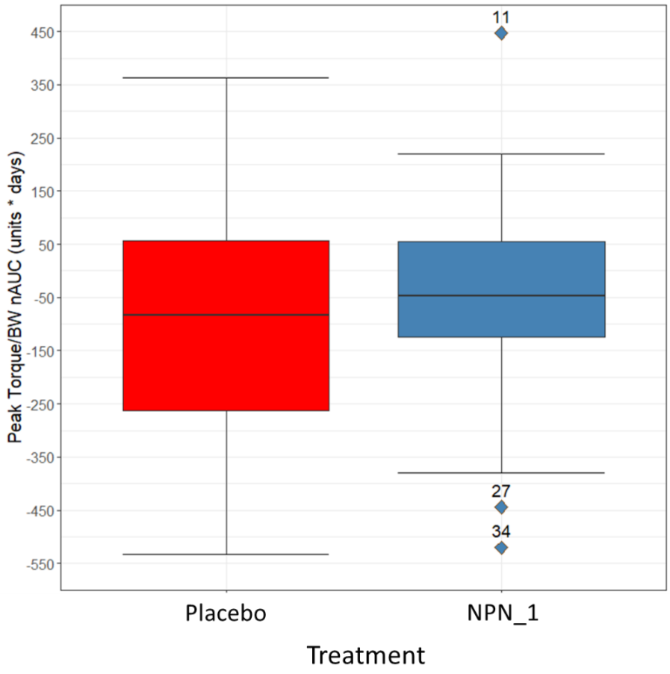

Figure S1. ROUT analysis for outlier identification. Boxplot representation of outliers as determined by ROUT analysis. Numbers indicate Participant IDs.

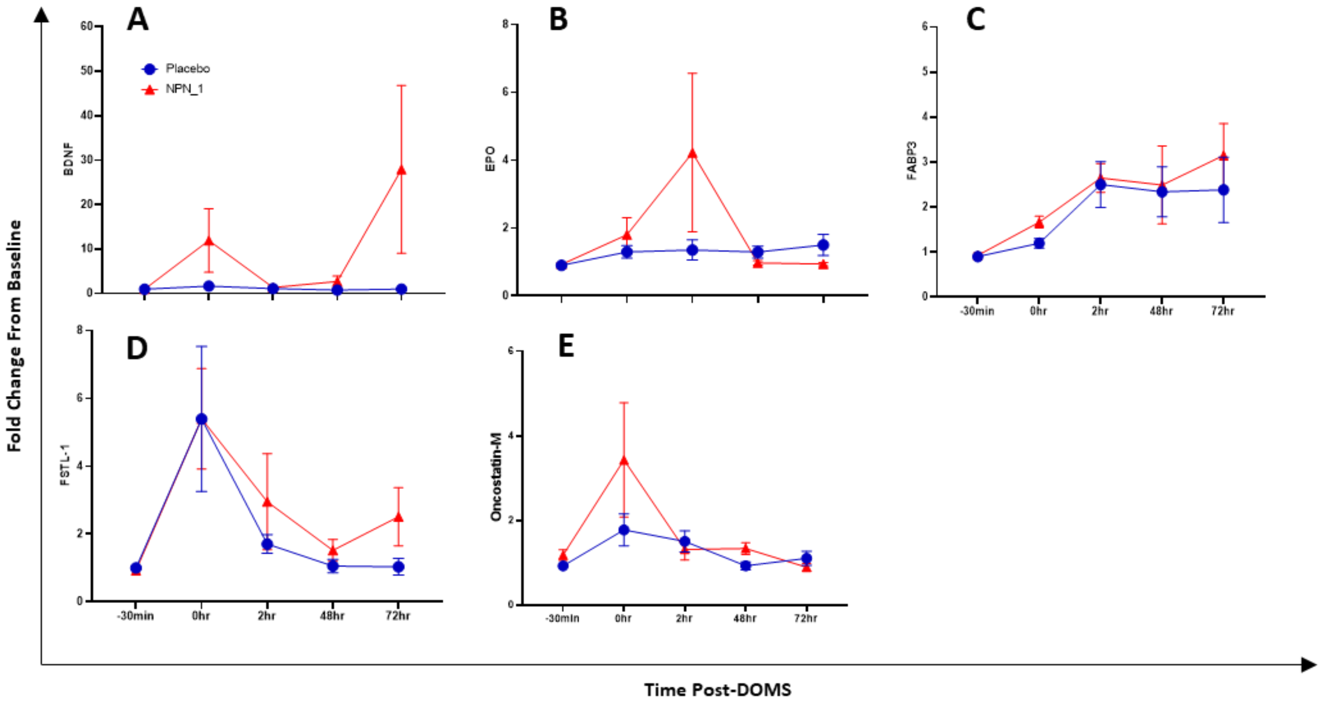

Figure S2. Supplementation with NPN_1 or placebo display similar effects on serum myokine release following intense exercise. Effect of supplementation on (A) BDNF, (B) erythropoietin (EPO), (C) FABP3, (D) FSTL-1 and (E) Oncostatin-M (Mean ± SEM).
